# A validated and standardized pseudotyped microneutralization assay as a safe and powerful tool to measure LASSA virus neutralising antibodies for vaccine development and comparison

**DOI:** 10.12688/f1000research.149578.2

**Published:** 2024-10-14

**Authors:** Roberta Antonelli, Vittoria Forconi, Eleonora Molesti, Claudia Semplici, Pietro Piu, Maria Altamura, Francesca Dapporto, Nigel Temperton, Emanuele Montomoli, Alessandro Manenti

**Affiliations:** 1 Vismederi, Siena, Italy, 53100, Italy; 2 Life-Science, University of Siena, Siena, Italy; 3 Viral Pseudotype Unit, Medway School of Pharmacy,, University of Kent and Greenwich at Medway, Chatham, Kent, UK; 4 Department of Molecular and Developmental Medicine, University of Siena, Siena, Italy

**Keywords:** Lassa virus (LASV), International Standard and Reference Panel for Anti-Lassa Fever, neutralisation assay, international guidelines

## Abstract

**Background:**

Over the past few decades, World Health Organization (WHO) has made massive efforts to promote the development of a vaccine against Lassa virus (LASV), one of the top ten priority pathogens for research and development under the WHO R&D Blueprint for Emerging Infections. To date, several vaccines are at different stages of development. In this scenario, a validated and standardised assay to measure LASV neutralising antibodies is urgently needed for vaccine development and comparison.

**Methods:**

The neutralisation assay remains the gold standard for determining antibody efficacy. Here we have proposed a safe and validated pseudotyped neutralisation assay for LASV, taking advantage of the development of the first WHO International Standard and Reference Panel for Anti-Lassa Fever (NIBSC code 21/332).

**Results and Conclusions:**

The proposed results demonstrate that the pseudotyped luciferase neutralisation assay is a specific serological test for the measurement of LASV neutralising antibodies without cross-reacting with standard sera specific for heterologous viral infections. In addition, the assay is accurate, precise, and linear according to criteria and statistical analyses defined and accepted by international guidelines.

## 1. Introduction

Lassa virus (LASV) is the causative agent of Lassa fever (LF), a zoonotic disease associated with an acute and potentially fatal haemorrhagic illness with approximately 100,000-300,000 human cases ^1^ and 5000 deaths per year. ^2^

First described in 1969 in Lassa, Nigeria, ^3^ LF is currently endemic in West and Central Africa, including Nigeria, Sierra Leone, Guinea, Liberia, Benin, Ghana and Mali and neighbouring countries. ^4^^,^^5^ Worldwide, sporadic cases of LASV infection have been reported in Europe, Japan, and the USA, imported by travellers from West Africa. ^6^^–^^8^

LASV is an enveloped-RNA virus with a genome consisting of two ambisense, single-stranded RNA segments, large (L) and small (S). Both RNA segments contain two open reading frames (ORFs) separated by non-coding intergenic regions (IGRs) and are involved in RNA transcription termination. ^1^ The L segment encodes the Z protein and RNA polymerase, while the S RNA segment encodes the nucleoprotein (NP) and the glycoprotein precursor complex (GPC), which is translationally cleaved co- and post- into GP1 and GP2 and the stable signal peptide (SSP). ^9^^,^^10^ Together, GP1, GP2 and SSP form the mature glycoprotein (GP) spike complex on the viral surface. GP1 is involved in receptor binding and entry into host cells, while GP2 and SSP are involved in stabilising receptor-GP complexes and in viral fusion within host cell membranes. GPC is the only viral protein on the surface of LASV and is the only target for neutralising antibodies (NAbs). ^11^

Typically, LASV is transmitted to humans through direct contact with its rodent reservoir, *Mastomys natalensis*, a multimammate rat, ^12^ which colonises rural domestic areas and promotes LASV spread via aerosolized excretions and secretions or consumption of contaminated food. ^13^ Less commonly, horizontal human-to-human transmission of LASV can occur, posing a higher risk to health care, which increases with disease progression and increasing viral load. ^14^^,^^15^ LASV infection is heterogeneous: 80% of infected individuals are asymptomatic or present with mild, non-specific symptoms; 20% of infected individuals present with severe symptoms, including haemorrhaging, respiratory distress, repeated vomiting, facial swelling, chest, back and abdominal pain, and shock. ^8^ Central nervous system manifestations and renal failure are strongly associated with a poor outcome ^16^; in the worst cases, death can occur within 14 days of symptom onset due to multiple organ failure. The case fatality rate (CFR) of Lassa fever, defined as the total number of deaths divided by the total number of officially diagnosed cases multiplied by 100, is estimated to be 15%, with wide variation between countries. To illustrate this variability, two recent studies of hospitalised populations in Nigeria reported CFRs of 14% and 31%, while a study in Sierra Leone estimated a CFR of 69%. ^17^^,^^18^

In 2016, due to its potential for zoonotic and human transmission, and difficulties in treatment and prevention, WHO designated LASV as one of the high priority pathogens identified in the WHO R&D Blueprint and by Coalition for Epidemic Preparedness Innovations (CEPI). The ultimate goal of this global health strategy is to promote research and development of medical countermeasures and vaccines for the treatment and control of LASV and all list of identified priority diseases in at-risk countries. ^19^

Improvement in the development of Lassa vaccines and treatments has been complicated by the designation of Lassa virus as a Category A pathogen by the National Institute of Allergy and Infectious Diseases (NIAID), and consequently Biosafety Level 4 (BSL-4) precautions are recommended for handling potentially infectious Lassa virus specimens. To overcome these limitations, pseudotyped viruses provide a powerful, safe, and convenient tool to investigate Lassa GP function, attachment, and entry processes; moreover, they are useful to analyse immunological responses and detect neutralising antibody. ^20^

Viral pseudotypes are chimeric non-replicating viruses that encode a reporter gene and carry the GP of interest on their surface. The laboratory use of pseudotyped virus particles has many advantages, particularly in the study of highly pathogenic viruses, as they can be handled in low-containment facilities, in tropism, drug screening, the screening and evaluation of monoclonal antibodies, vaccine evaluation and serosurveillance studies. ^21^^,^^22^

Neutralization assays are useful tools to study antibody responses after natural exposure to viruses, or responses elicited by vaccination. These assays normally require the application of wild-type viruses, which can be limiting if the virus in use is highly pathogenic in humans. ^23^^,^^24^

However, LASV pseudotypes have been used in various publications, none of which have previously described their applicability in a neutralisation assay. ^25^^,^^26^ In this study, we describe the successful validation of a safe pseudotyped virus-based neutralisation assay using serum samples from the first international reference panel for anti-Lassa fever virus antibodies provided by the Medicines and Healthcare products Regulatory Agency (MHRA). The present study, conducted according to International Council for Harmonisation of Technical Requirements for Pharmaceuticals for Human Use (ICH) guidelines ( https://www.ich.org/page/ich-guidelines), underlines the importance of the pseudotype platform as a tool to increase the safety and accessibility of potency testing, to support the establishment of international standards and reference panels together with live viruses, and to support vaccine clinical trials by monitoring sera from vaccine candidates.

## 2. Methods

### 2.1 Cell line maintenance

For the production and titration of Lassa virus pseudotypes, as well as for pseudotype-based microneutralization assay, human embryonic kidney 293T/17 (HEK293T/17) (ATCC, cat number CRL-11268) are maintained in complete medium Dulbecco’s modified essential medium (DMEM) high glucose, pyruvate (Gibco, Catalog number: 11995073). The medium is supplemented with 10% FBS South America Origin EU approved Heat Inactivated (Euroclone, Catalog number: ECS5000LH) and 1% penicillin–streptomycin (Pen-Strep) (Merk, cat number: P4333). The cells are kept in incubator at 37°C with 5% CO _2_.

### 2.2 Lassa virus and lentiviral vector packaging plasmids

The gene of Lassa virus (LASV) (strain Josiah) glycoprotein is synthesized and cloned into plasmid expression vector phCMV3 (Sino Biological, cat number: VG40079-UT). The p8.91 lentiviral packaging plasmid expressing *gag-pol*, and the pCSFLW plasmid containing a firefly luciferase reporter are provide by Prof. Nigel Temperton (Viral Pseudotype Unit-VPU, University of Kent Medway School of Pharmacy).

### 2.3 Pseudotype production and harvest

For LASV pseudotype production, approximately 4.5×10 ^6^ HEK293T/17 cells. 24h hours post seeding, cells are transfected with a plasmid DNA mixture composed of: 3 μg of p8.91, 4.5 μg of pCSFLW and 3 μg of LASV-G using 24 μl of EndoFectin™-Lenti (GeneCopoeia, Catalog number: EF001) in 500 μl reduced serum medium Opti-MEM 1X (Gibco, Catalog number: 31985-070). At least 6 hours post-transfection 8 ml of fresh DMEM is replaced to the plates. 72 hours post-transfection LASV pseudotype particles are collected, centrifuged, filtered through 0.45μm syringe-driven membrane filters and stored at -80°C.

### 2.4 Pseudotype titration assay

Pseudotype titration is performed in 96 well plate format: 50 μL of DMEM are added to every well except raw A where 100 μL of LASV pseudotype particles are added. Serial two-fold dilutions are performed from raw A to G, and 1×10 ^5^ HEK293T/17 cells are added to each well. After 48 hours, 50 μL of Bright-Glo Luciferase Assay System (Promega, catalog number: E2620) are dispensed in each well and placed in a microplate shaker for 5 minutes at 400 rpm (Fisherbrand Catalog number: 88861024). Plate readout is performed with luminometer (Perkin Elmer; Victor Nivo) following parameter: Luminescence Endpoint, counts single label, 700 nm, 1000 ms measurement time.

### 2.5 Pseudotype based microneutralization assay

Pseudotype-based microneutralization (PBNA) is performed in 96 well plate format. LASV pseudotype working solution corresponds to 1×10 ^8^ relative luminescence unit (RLU) divided by the pseudotype titre expressed in RLU/mL. 90 μL of DMEM are added to column one and 50 μL are added to the other wells. To column 1 are added 10 μL of serum samples (1st International Reference Panel for anti-Lassa fever virus antibodies 21/332 NIBSC containing 20/228, 20/204, 20/246, 20/222 and 20/248) at the proper dilution and immunoglobulins depleted serum (BBI Solutions, Catalog number: SF505-2) as used as negative control. Serial two-fold dilutions are performed from column 1 to 10. Subsequently, 50 μL of pseudotype working solution are added to each well up to column 10, and to column 12 from raw A to H (pseudotype control wells). The plate containing the mix pseudotypes-serum is incubated for 1 hour at 37°C with 5% CO _2_, afterwards 1×10 ^5^ HEK293T/17 cells are added to each well. After 48 hours, 50 μL of Bright-Glo Luciferase Assay System are dispensed in each well and the plate is shake for 5 minutes at 400 rpm in a dark environment (Fisherbrand, Catalog number: 88861024). Plate readout is performed with a luminometer (Perkin Elmer; Victor Nivo).

**Table 1.** Observed and expected geometric means, relative accuracy for each control serum and dilution.

20/228 High ctr LASSA				
Fold Dilution	Observed GMT	Expected GMT	Relative Accuracy (RA)	50% ≤ RA ≤ 200%
1	558.74	558.74	100.00%	**PASSED**
2	432.42	279.37	154.78%	**PASSED**
4	261.36	139.69	187.11%	**PASSED**
8	97.88	69.84	140.14%	**PASSED**
16	23.98	34.92	68.68%	**PASSED**
32	10.00	17.46	57.27%	**PASSED**
64	10.00	8.73	114.54%	**PASSED**

20/204 Middle ctr LASSA				
Fold Dilution	Observed GMT	Expected GMT	Relative Accuracy (RA)	50% ≤ RA ≤ 200%
1	373.49	373.49	100.00%	**PASSED**
2	267.61	186.74	143.30%	**PASSED**
4	154.66	93.37	165.64%	**PASSED**
8	34.79	46.69	74.51%	**PASSED**
16	10.00	23.34	42.84%	**FAILED**
32	10.00	11.67	85.68%	**PASSED**
64	10.00	5.84	171.36%	**PASSED**

20/222 Low ctr LASSA				
Fold Dilution	Observed GMT	Expected GMT	Relative Accuracy (RA)	50% ≤ RA ≤ 200%
1	81.67	81.67	100.00%	**PASSED**
2	53.34	40.83	130.62%	**PASSED**
4	10.00	20.42	48.98%	**FAILED**
8	10.00	10.21	97.96%	**PASSED**
16	10.00	5.10	195.92%	**PASSED**

### 2.6 Statistical analysis

Calculation of Pseudotype titres were estimated by means of Excel ^TM^ software; the pseudotype titres obtained at each point in a range of dilution points were expressed as RLU/mL, and the arithmetic mean was calculated. For the analyses of pseudotype-based neutralisation assays, the GraphPad Prism version 8.4 package was used (GraphPad Software, GraphPad, 2365 Northside Dr, Suite 560, San Diego, CA 92108, USA). The titres were firstly normalised based on pseudotype only mean value and cell only mean value, and IC _50_ values were calculated by a non-linear regression model (log (inhibitor) vs. normalised response-variable slope) analysis. Titres were subsequently expressed as the range of dilution in which the IC _50_ value lay. ^27^

### 2.7 Relative accuracy

The assessment of the accuracy of a test is conducted by analyzing the data acquired for the evaluation of Dilutional Linearity (GMTs observed). According to the ICH guideline Q2 (European Medicines Agency. ICH Q2(R2) Validation of analytical procedures - Scientific guideline. 2022 ^28^), there are two ways to test the accuracy of a test: by using a conventional true value or an accepted reference value as the expected value of GMT. The expected GMTs are determined by calculating the geometric mean of the results obtained from the neat sample divided by the corresponding factor of the two-fold serial dilution. To evaluate the Relative Accuracy, the percentage of recovery on the GMT of the observed values and the expected (true) titre is calculated by applying the following formula:

$$100∙\left(\frac{{\mathrm{GMT}}_{\text{observed}}}{{\mathrm{GMT}}_{\text{expected}}}\right)$$

### 2.8 Dilutional linearity

Linearity is assessed by testing the 20/228, the 20/222 and the 20/204 samples from NIBSC reference panel for anti-Lassa fever virus in a two-fold dilution scheme, starting from a dilution of 1:20 to 1:2560 dilution The sample dilutions were tested in two repetitions per plate, and in two different plates by two different operators, on two different days. For each dilution, the geometric mean titre (GMT) is calculated. A linear regression analysis of logarithm base 2 of serum dilution versus logarithm base 2 of GMTs using the least squares method over the entire dilution range is performed and the slope of regression line and the coefficient of determination (R ^2^) is calculated.

### 2.9 Precision

Precision is used to define the amount of scatter between several measurements obtained from multiple testing of the same sample under the standardized conditions. The Precision parameters are evaluated by the Percent Geometric Coefficient of Variation (%GCV) determined as

$$\%\mathrm{GCV}=100∙\left(2^{\sigma }-1\right)$$

where σ is the standard deviation of log-transformed (base 2) data. To assess the Precision validation parameters the data of Dilutional Linearity experiments are used.

The validation process considers four crucial aspects of precision that are essential to evaluate the reliability of laboratory testing.

These aspects include intra-day variation, which measures the expected variation within each day under identical operating conditions.

Additionally, intra-operator variation is evaluated, which measures the expected variation within each operator under identical operating conditions.

Intermediate precision, also known as inter-test variation, is considered to account for the variations and random events that may occur within the laboratory due to different environmental conditions, operators, equipment, or days.

A further aspect is format variability (FV), which evaluates the expected variation between the average titer results obtained from multiple replicates in routine testing and considers two independent analyses (k = 2), each consisting of one replicate (n = 1). The FV is determined by the following equation:

$$\mathrm{FV}=2^{\sqrt{\frac{\sigma_{\mathrm{IR}}^{2}}{k}+\frac{\sigma_{R}^{2}}{\mathrm{nk}}}}-1$$

where $\sigma_{\mathrm{IR}}^{2}$ is the between-run (or inter-run) variance component and $\sigma_{R}^{2}$ the within-run (or residual) component. The total variance is the sum of the between- and within-run components $\sigma_{T}^{2}=\sigma_{\mathrm{IR}}^{2}+\sigma_{R}^{2}$ .

### 2.10 Specificity

An assay is defined as specific when it can, in a non-ambiguous manner, detect the analyte in the presence of other components. Here, specificity was assessed by testing LASV pseudotype against the second International Standard for anti-SARS-CoV-2 immunoglobulin (NIBSC code: 21/340), and against the first WHO International Reference Panel of Anti-Ebola virus (EBOV) Convalescent Plasmas (NIBSC code: 16/344) whose individual panel members are reported with the following NIBSC codes: 15/280 (ARC), 15/282 (NHSBT), 15/284 (NOR), 15/286 (INMI), 15/288 (negative human plasma). As negative control the immunoglobulin’s depleted serum was used (“Pseudotype based microneutralization assay” paragraph).

To assess the Specificity parameter the ratio between sera and no specific NIBSC, and between sera and negative samples is calculated.

## 3. Results

### 3.1 Lassa envelope glycoprotein pseudotyped lentivirus particles

**Figure 1.**
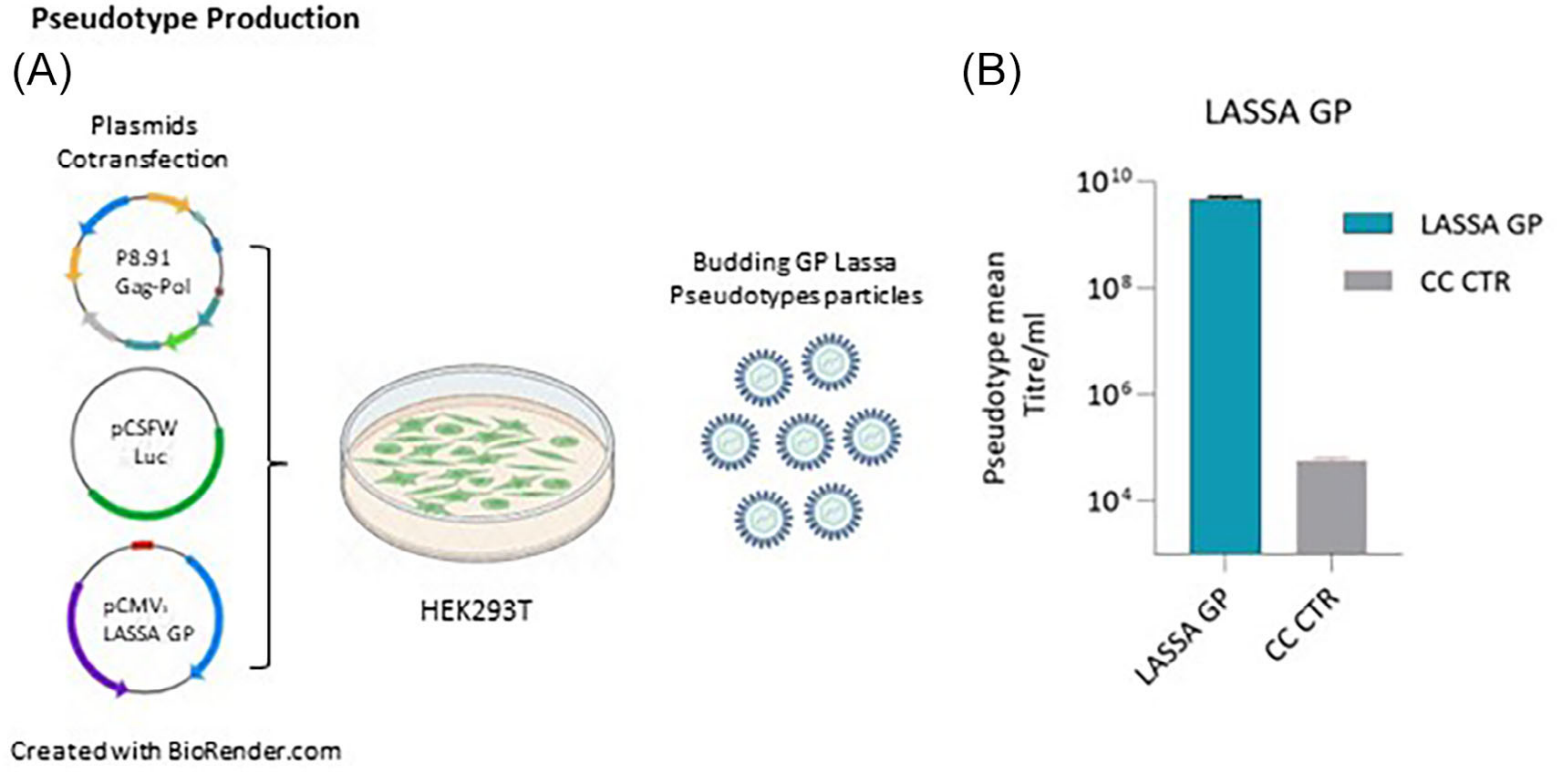
Schematic representation describing PV production and titration. (A) Packaging construct gag pol, reporter vector and pCMV3-GP LASV (strain Josiah) expression plasmids are co-transfected into HEK293/17 cells. Pseudotype proteins are packaged by the cell and budding occurs at the cell membrane to yield pseudotypes bearing desired glycoproteins and incorporated reporter. Culture supernatant was collected 72 hours after transfection and filtered through a 0.45 um filter. (B) Target HEK293/17 cells were transduced with LASV GP-pseudotyped lentiviral particles and the infectivity measured as relative luminescent units (RLU/mL). The grey column indicated the cellular control (CC CTR) as comparison.

To develop a safe and robust pseudotype neutralisation assay for LASV, phCMV3-GP LASV (strain Josiah) was co-transfected with p.8.91 gag-pol plasmid and pCSFLW plasmid containing a firefly luciferase reporter as shown in Figure 1A. 72 h post-transfection, cell culture supernatants containing pseudotypes particles (PV) were harvested and their transduction efficiency was assessed by measuring titres expressed as RLU/ml in HEK293/17 cells. As shown in Figure 1B, Lassa GP showed a pseudotype mean titre/ml of 4.98e+9 RLU/ml.

### 3.2 *In vitro* inhibition of LASV pseudotypes by antisera

To validate this pseudotype neutralisation assay for LASV, we performed a series of validation experiments using the MHRA Lassa panel 21/332 (1st International Reference Panel for anti-Lassa fever virus antibodies 21/332 NIBSC ).

**Figure 2.**
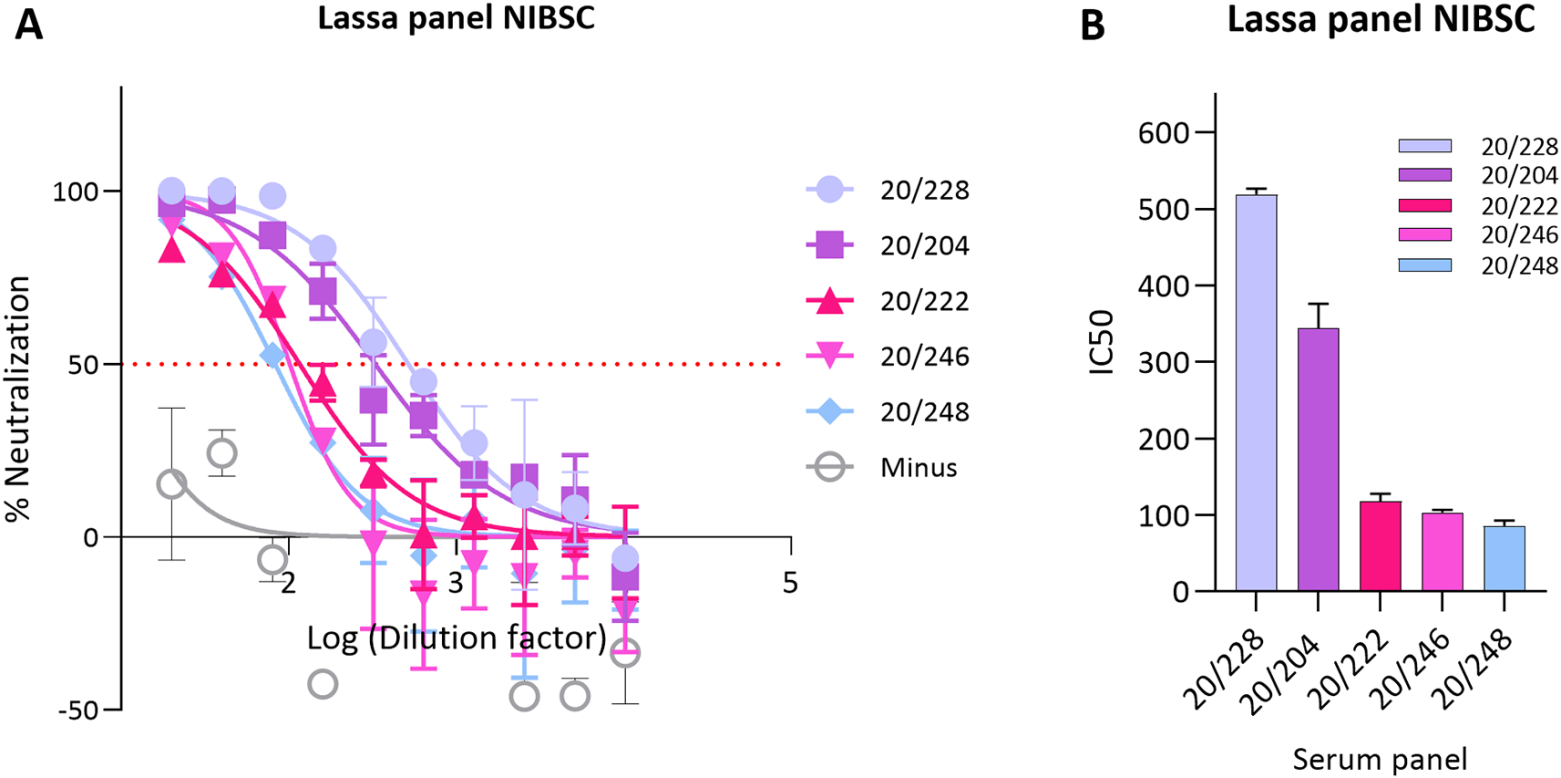
*In vitro* inhibition of pseudotypes by Lassa panel 21/332 antisera. (A) Neutralisation curves obtained from WHO Lassa panel. Reference antisera were used at starting dilution of 1:20 while LASV PV diluted to 1.000.000 RFU dose input as determined previously from titration. Each points represents the mean and standard deviation of two replicates per dilution repeated for three independent experiments. (B) Half-maximal inhibitory dilution of reference antisera as calculated from “log (inhibitor) vs. normalized response—Variable slope” from “Dose-response-Inhibition using Graph pad software. The values are previously normalized on only mean value of PV value and cell-only mean value.

This panel consisted of 5 sera (20/228, 20/204, 20/222, 20/246 and 20/248) and was established by an expert committee on biological standardisation in 2021. First, the IC50 of all 5 sera in the panel were tested against LASV GP pseudotypes/plate and 20/228 was selected as high control, 20/204 as medium and 20/222 as low control, Figure 2.

### 3.3 Relative accuracy

To evaluate the relative accuracy of the assay, each serum was tested in 2 different analytical sessions performed by 2 operators with 2 replicates per session. The assay is considered to have acceptable relative accuracy if the percentage of recovery on the GM of the reportable values and the expected is within 50% - 200%. The assay is accurate from 1 to 64 dilutions for 20/228 standard. In addiction the table below, Table 1, also shows that the RAs at the 1:16 dilution for 20/204 and the 1:4 dilution for 20/222 are just below 50% but can be considered within the range of accurate dilutions as they are between multiple accurate dilutions.

### 3.4 Precision

**Figure 3.**
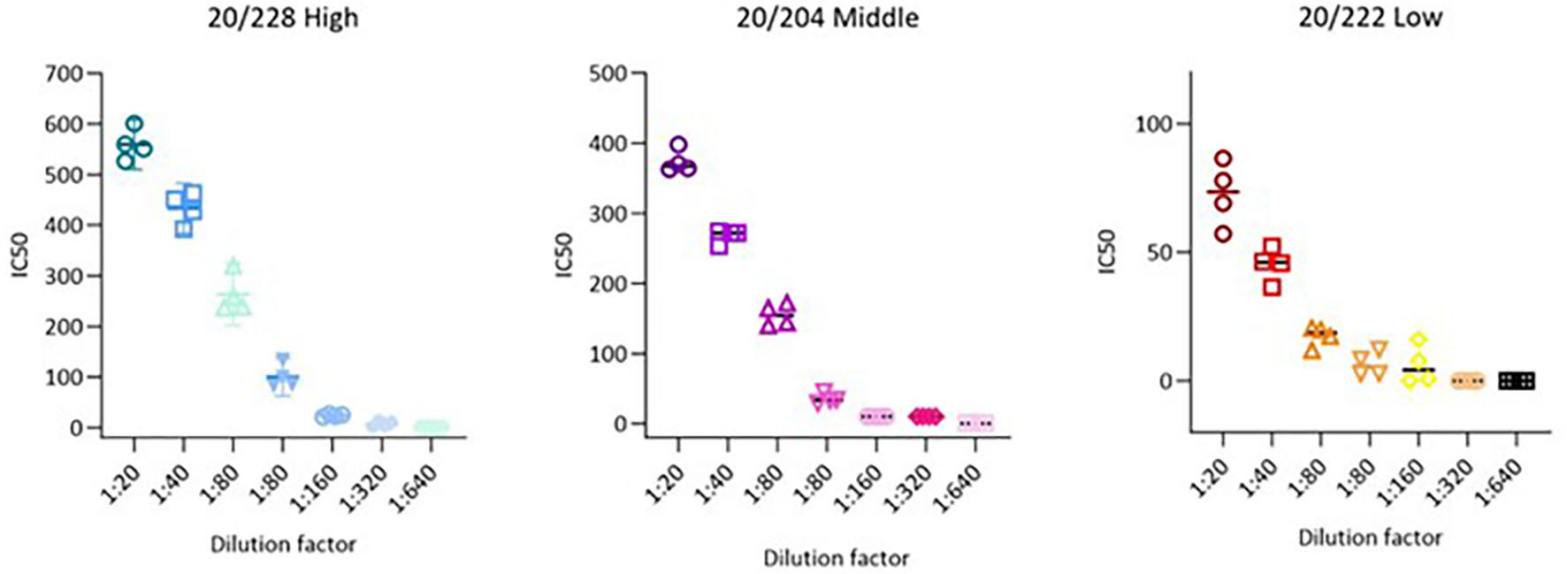
Precision representation for all dilution for 20/228 high, 20/204 middle and 20/222 low serum. Visual plot of the precision results obtained from each analysed sample. The x-axis shows the different dilutions, while the y-axis shows the IC50 value. Each dot represents one replicate, performed in a different day or by a different operator. Straight lines indicate the mean of the 4 values, coupled with the SD of the replicates.

Precision parameters are usually evaluated through the variance, standard deviation, or coefficient of variation of a series of measurements, Figure 3.

**3.4.1 Intra-Day variation**

The two geometric means between results from same day but different operator (D1-O1 and D1-O2, D2-O1 and D2-O2) are calculated for each dilution. The Intra-Day variation is determined by the %GCV of the two geometric means.

The assay is considered to have acceptable Intra-Day variation if all samples have a %GCV less or equal than 50%. The results shown in Table 2 indicate that the assay has acceptable Intra-Day variation from 1:20 (neat) to 1:2560 for 20/228, 20/224 and 20/222.

**Table 2.** Intra-day variation estimates in log transformation.

Intra-Day variation									
	20/228			20/204			20/222		
Dilution	SD	%GCV	≤50%	SD	%GCV	≤50%	SD	%GCV	≤50%
1:20 (neat)	0.06	4.20%	**PASSED**	0.04	2.71%	**PASSED**	0.15	11.05%	**PASSED**
1:40	0.03	2.13%	**PASSED**	0.04	2.86%	**PASSED**	0.12	8.43%	**PASSED**
1:80	0.19	14.34%	**PASSED**	0.04	2.50%	**PASSED**	0.00	0.00%	**PASSED**
1:160	0.32	25.22%	**PASSED**	0.21	15.58%	**PASSED**	0.00	0.00%	**PASSED**
1:320	0.08	5.56%	**PASSED**	0.00	0.00%	**PASSED**	0.00	0.00%	**PASSED**
1:640	0.00	0.00%	**PASSED**	0.00	0.00%	**PASSED**	0.00	0.00%	**PASSED**
1:1280	0.00	0.00%	**PASSED**	0.00	0.00%	**PASSED**	0.00	0.00%	**PASSED**
1:2560	0.00	0.00%	**PASSED**	0.00	0.00%	**PASSED**	0.00	0.00%	**PASSED**

The two geometric means between results from same operator but different day (D1-O1 and D2-O1, D1-O2 and D2-O2) are calculated for each dilution. The Intra-Operator variation is determined by the %GCV of the two geometric means.

The assay is considered to have acceptable Intra-Operator variation if all samples have a %GCV less or equal than 50%. The assay has also acceptable Intra-Operator variation from 1:20 (neat) to 1:2560 for 20/228, 20/224 and 20/222 ( Figure 3, Table 3).

**Table 3.** Intra-operator variation estimates in log transformation.

Intra-operator variation									
	20/228			20/204			20/222		
Dilution	SD	%GCV	≤50%	SD	%GCV	≤50%	SD	%GCV	≤50%
1:20 (neat)	0.08	5.40%	**PASSED**	0.06	3.91%	**PASSED**	0.10	7.33%	**PASSED**
1:40	0.11	8.15%	**PASSED**	0.04	2.86%	**PASSED**	0.19	13.95%	**PASSED**
1:80	0.11	8.15%	**PASSED**	0.01	0.47%	**PASSED**	0.00	0.00%	**PASSED**
1:160	0.13	9.60%	**PASSED**	0.08	5.49%	**PASSED**	0.00	0.00%	**PASSED**
1:320	0.00	0.03%	**PASSED**	0.00	0.00%	**PASSED**	0.00	0.00%	**PASSED**
1:640	0.00	0.00%	**PASSED**	0.00	0.00%	**PASSED**	0.00	0.00%	**PASSED**
1:1280	0.00	0.00%	**PASSED**	0.00	0.00%	**PASSED**	0.00	0.00%	**PASSED**
1:2560	0.00	0.00%	**PASSED**	0.00	0.00%	**PASSED**	0.00	0.00%	**PASSED**

**3.4.2 Intra-Operator variation**

The two geometric means between results from same operator but different day (D1-O1 and D2-O1, D1-O2 and D2-O2) are calculated for each dilution. The Intra-Operator variation is determined by the %GCV of the two geometric means.

The assay is considered to have acceptable Intra-Operator variation if all samples have a %GCV less or equal than 50%. The assay has also acceptable Intra-Operator variation from 1:20 (neat) to 1:2560 for 20/228, 20/224 and 20/222 ( Figure 3, Table 3).

**3.4.3 Intermediate precision**

The Intermediate Precision (IP) is assessed by calculating the %GCV of the four determinations for each dilution. The assay is deemed to be precise if all estimates of intermediate precision have %GCV less or equal than 50%. The results reported in Table 4 demonstrate that the assay is precise from 1:20 (neat) to 1:2560 for high, medium, and low serum.

**Table 4.** Intermediate precision estimates in log transformation log-transformation.

Intermediate precision									
	20/228			20/204			20/222		
Dilution	SD	%GCV	≤50%	SD	%GCV	≤50%	SD	%GCV	≤50%
1:20 (neat)	0.08	5.64%	**PASSED**	0.06	4.42%	**PASSED**	0.15	11.05%	**PASSED**
1:40	0.11	7.64%	**PASSED**	0.05	3.83%	**PASSED**	0.21	15.80%	**PASSED**
1:80	0.20	15.07%	**PASSED**	0.15	10.61%	**PASSED**	0.00	0.00%	**PASSED**
1:160	0.32	24.62%	**PASSED**	0.27	20.32%	**PASSED**	0.00	0.00%	**PASSED**
1:320	0.15	10.77%	**PASSED**	0.00	0.00%	**PASSED**	0.00	0.00%	**PASSED**
1:640	0.00	0.00%	**PASSED**	0.00	0.00%	**PASSED**	0.00	0.00%	**PASSED**
1:1280	0.00	0.00%	**PASSED**	0.00	0.00%	**PASSED**	0.00	0.00%	**PASSED**
1:2560	0.00	0.00%	**PASSED**	0.00	0.00%	**PASSED**	0.00	0.00%	**PASSED**

**3.4.4 Format variability**

The Format Variability (FV) measures the variability of a bioassay and is expressed as %GCV. The FV has a strong relationship with Critical Fold Difference (CFD) parameter given by $\mathrm{CFD}={\left(1+\mathrm{FV}\right)}^{q_{\alpha }}$ , where qα is the quantile from the t-distribution with α = 0.01 and infinite degrees of freedom, which is well and properly approximated by the standard normal percentile zα = 2.326348. CFD is set to 4, and therefore the assay will be considered to have acceptable FV if all samples met %GCV ≤ 81.5%. The results shown in Table 5 indicate that the assay has acceptable FV from 1:20 (neat) to 1:2560 dilution for all control sera.

**Table 5.** Format variability %GCVs for each control serum and dilution.

Format variability						
	20/228		20/204		20/222	
Dilution	FV	≤81.5%	FV	≤81.5%	FV	≤81.5%
1:20 (neat)	12.35%	**PASSED**	10.85%	**PASSED**	17.70%	**PASSED**
1:40	14.51%	**PASSED**	10.07%	**PASSED**	21.51%	**PASSED**
1:80	20.96%	**PASSED**	17.31%	**PASSED**	0.00%	**PASSED**
1:160	27.53%	**PASSED**	24.73%	**PASSED**	0.00%	**PASSED**
1:320	17.45%	**PASSED**	0.00%	**PASSED**	0.00%	**PASSED**
1:640	0.00%	**PASSED**	0.00%	**PASSED**	0.00%	**PASSED**
1:1280	0.00%	**PASSED**	0.00%	**PASSED**	0.00%	**PASSED**
1:2560	0.00%	**PASSED**	0.00%	**PASSED**	0.00%	**PASSED**

### 3.5 Linearity

**Figure 4.**
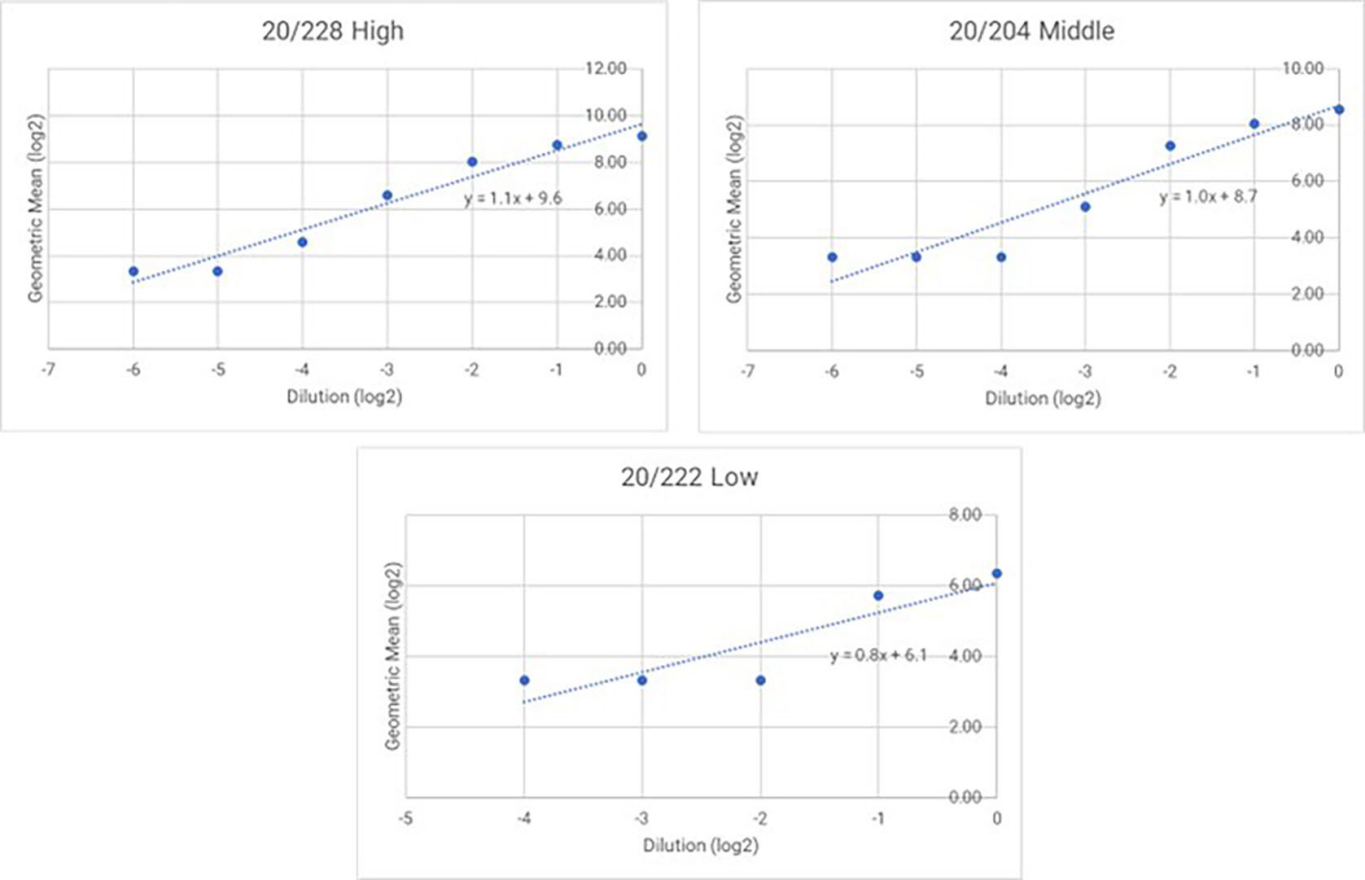
Linear regression lines for 20/228 high, 20/204 middle and 20/222. The proposed graph show linear regression slope for 20/228 high, 20/204 medium and 20/222 Lassa serum. Logarithm (base 2) of the dilution is indicated on the x-axis and the logarithm (base 2) of the obtained GMT on the y-axis.

**Figure 5.**
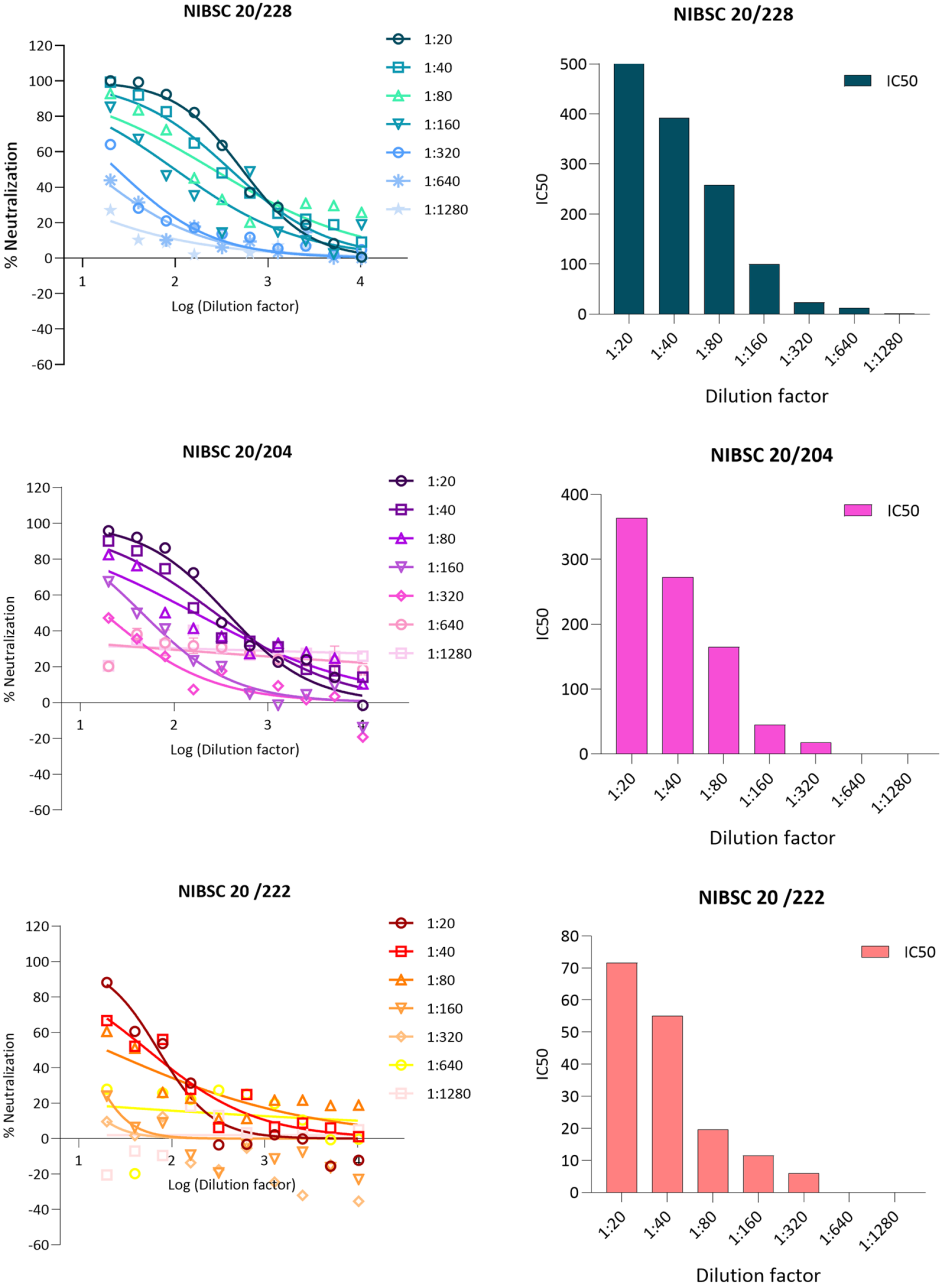
In-vitro inhibition of Lassa panel 21/332 (20/228, 20/224 and 20/222) antisera at different serum serial starting dilution. Dots represent % neutralisation average between the 4 replicates, error bars indicate standard deviation. Neutralization curves of serially diluted two-fold from a starting dilution of 1:20 to 1:2560. For all plots, each point represents the mean of two replicates per dilution.

Dilution linearity is a critical evaluation conducted to ensure that a sample with a high concentration can be diluted to a concentration within the working range and still provide a reliable result. This test determines the linearity of the dose-response of the analyte, which is fundamental to ensuring that sample dilution does not affect accuracy and precision. Acceptable linearity of the test is achieved when the absolute value of the slope falls within the range of 0.7 to 1.3. The absolute slope values of samples 20/228, 20/204, and 20/222 were 1.1, 1.0, and 0.8, respectively as showed in Figure 4 and Figure 5.

### 3.6 Specificity

**Figure 6.**
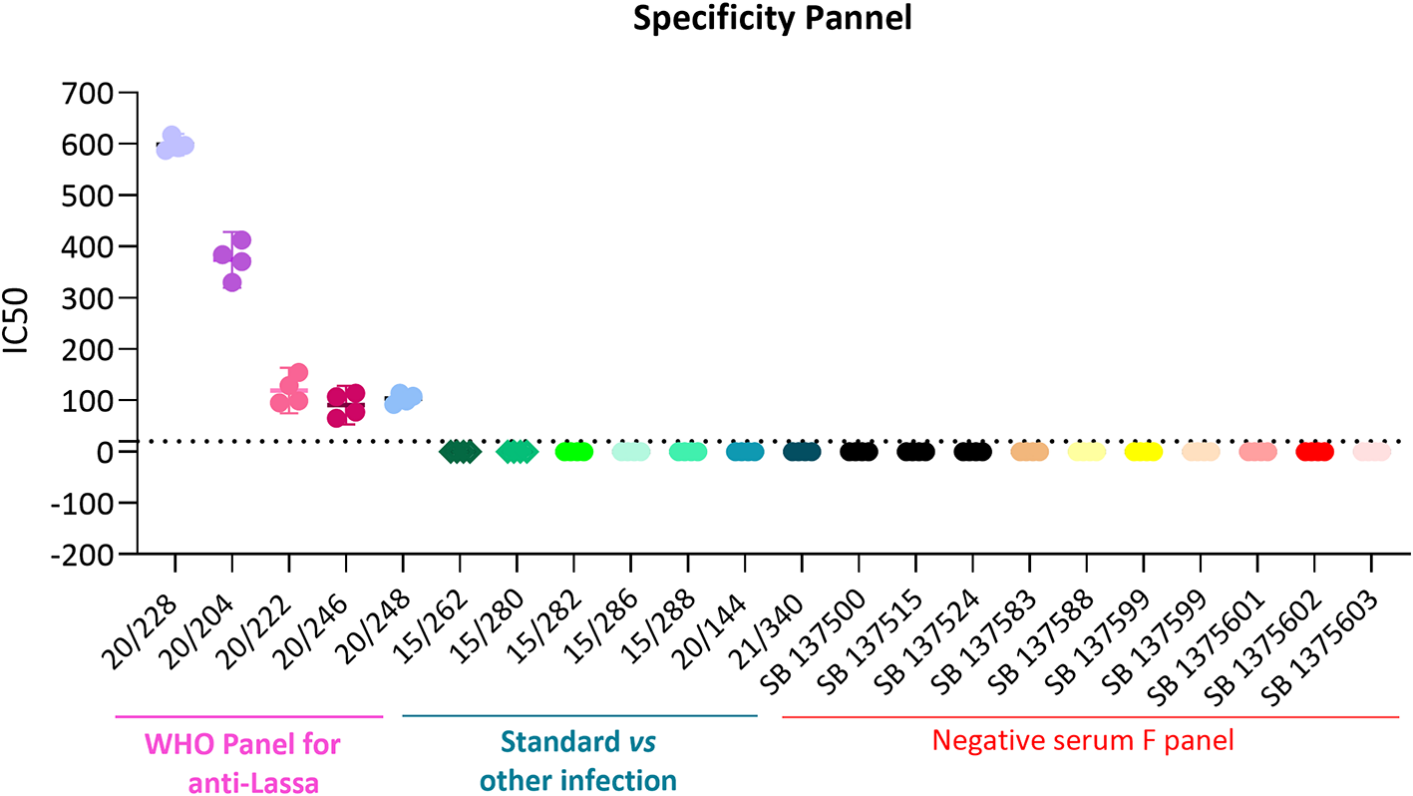
Specificity results for the full Lassa panel: 20/228, 20/204, 20/222, 20/246 and 20/202. All 5 sera from the Lassa panel, positive versus heterologous viruses and negative donors were tested against LASV pseudoviruses as indicated in x axes. Each symbol represents a single IC50 value for the different sera, Each serum was tested in duplicated in two different days.

Specificity is the ability of the assay to detect and differentiate the analyte of interest. ^23^^,^^24^^,^^27^ To determine the specificity of the assay, all 5 sera from the Lassa panel, positive versus heterologous viruses and negative donors were tested. As visualised in Figure 6 and Table 6, 20/228 showed a fold difference of 56 versus heterologous virus serum and negative donors; 20/204 a fold difference of 37; 20/222 a fold difference of 16; 20/246 a fold difference of 8 and 20/248 a fold difference of 5. All 5 components of the panel are specific for this assay.

**Table 6.** Specificity fold-change.

	Heterologous panel									
fold-change	ebola 15/262	ebola 15/280	ebola 15/282	ebola 15/284	ebola 15/286	15/288	20/186	20/144	21/340	minus
20/228	56	56	56	56	56	56	56	56	56	56
20/204	37	37	37	37	37	37	37	37	37	37
20/222	16	16	16	16	16	16	16	16	16	16
20/246	8	8	8	8	8	8	8	8	8	8
20/248	5	5	5	5	5	5	5	5	5	5

	Negative panel									
fold-change	SB 137500	SB 137515	SB 137517	SB 137524	SB 137583	SB 137588	SB 137599	SB 137601	SB 137602	SB 137606
20/228	56	56	56	56	56	56	56	56	56	56
20/204	37	37	37	37	37	37	37	37	37	37
20/222	16	16	16	16	16	16	16	16	16	16
20/246	8	8	8	8	8	8	8	8	8	8
20/248	5	5	5	5	5	5	5	5	5	5

The assay will be considered specific if Lassa serum have at least a four-fold difference (i.e., ≥ 4-fold) to the heterologous panel and negative panel.

## 4. Discussion

The emergence of coronavirus disease 2019 (COVID-19) has had a variety of devastating public health and socioeconomic impacts around the world, leading to a decline in the epidemiological control of several infectious diseases, including zoonotic diseases such as Lassa fever LF. ^29^

In recent years, epidemiological data show that the magnitude and timing of Lassa fever (LF) outbreaks have increased, particularly in Nigeria, ^30^ where the case fatality rate (CFR) continues to hover around 20%. ^31^^,^^32^ In endemic areas, the annual incidence of Lassa virus (LASV) infection ranges from 100,000 to 300,000 cases and the associated mortality is between 5,000 and 10,000 deaths per year. ^33^^,^^34^ However, challenges with clinical diagnosis and limited existing surveillance infrastructure mean that the disease is likely to be significantly under-reported and that incidence rates in countries where the disease is known to be endemic are imprecise. Due to its epidemic potential, combined with no or inadequate control measures, LASV has been identified as one of the top ten priority pathogens for research and development under the WHO R&D Blueprint for Emerging Infections. ^35^

The overall goal of the R&D Blueprint is to reduce the time needed to develop safe and effective medical countermeasures - both curative and preventive - and to accelerate the development of diagnostic treatments and vaccines. ^34^ To achieve this goal, several vaccines against LASV are in development as a result of global efforts, six of which are supported by the CEPI. In parallel, the development of biological standards and validated assays will be critical to assess vaccine-induced immune responses and promote standardisation, transparency, and comparability between candidates. ^36^ In this scenario, a standardised neutralisation assay will facilitate the down-selection of different vaccine candidates for clinical development, as the neutralisation titre is a key parameter for predicting immunity, i.e. for vaccine development. ^37^

Pseudovirus-based neutralisation assays (PBNAs) are a powerful tool extensively used in vaccine development - in fact, they have been used in several preclinical studies to evaluate vaccine candidates, assess the neutralising capacity of vaccine-induced antibodies, monitor vaccine efficacy against evolving virus variants, and accelerate the development of critical countermeasures against major diseases. ^36^

Several examples of the use of PBNAs for vaccine development can be highlighted: in vitro vaccine efficacy studies have been successfully performed using pseudotyped H7N9 virus, ^37^ Rift Valley fever virus, ^38^ Nipah virus, ^39^ rabies virus ^20^ and chikungunya virus. ^40^ PBNAs have also been useful in studying the immunogenicity of HPV vaccines such as CervarixTM and Gardasil. ^41^^,^^42^ In the recent COVID-19 pandemic, PBNAs played a key role in screening for cross-neutralising antibodies, ^43^ evaluating monoclonal antibodies and assessing vaccine titers, which is particularly important in the context of emerging and mutated variants of the virus. ^44^

In October 2018, WHO endorsed the production of an international standard for anti-Lassa virus antibodies to establish a reference material that would allow comparison of LASV serological data reported between laboratories and at different stages of clinical trials. In this collaborative study, pools of convalescent sera or plasma from either Sierra Leone or Nigeria with different anti-LASV antibody titres, including a negative control, were tested by participants using serological assays routinely used in their laboratories. Most participants, including the serology department of Vismederi srl, used the Josiah live virus isolate or GPC and NP proteins derived from the Josiah LASV isolate. Finally, in 2021, the collaborative study (WHO/BS/2021_2406) enabled the establishment of the first WHO International Standard and Reference Panel for Anti-Lassa Fever (NIBSC code 21/332), a valuable tool for the development and evaluation of the sensitivity of serological assays for LASV antibodies. ^45^

Taking advantage of the availability of this international anti-Lassa fever reference panel and following the efficient production of the LASSA GPC pseudotype by adapting the protocol described in Ferrara and Temperton 2018, we developed a lentiviral pseudotype-based assay to facilitate the accurate determination of neutralising antibody responses to LASV.

As previously anticipated PBNA are a valid tool that can be used as a surrogate for live viruses to study host-virus interactions and to test the ability of a given serum sample to neutralise virus particles in vitro. For the latter, validation and/or qualification procedures play a key role in providing reliable results, especially when they can be used to evaluate the efficacy of new potential vaccines or antiviral drugs. It is important that experiments, criteria, and statistical analyses follow defined and accepted international guidelines (European Medicines Agency. ICH Q2(R2) Validation of analytical procedures - Scientific guideline. 2022 ^28^).

Here we performed a series of optimisation and validation experiments of the PBNA and panel 21/332, which allowed us to establish the best experimental conditions to minimise the variation in data obtained from different experiments. Specifically, we performed the accuracy, precision, and linearity on three sera from the panel: 20/228, which according to the report gave us the highest titer samples for neutralising antibodies, 20/204, which showed medium titer neutralising antibodies, and 20/222, one of the three lowest titer samples in the whole panel. To evaluate the specificity parameter, we used all five sera in the LASSA panel, 7 serum-positive heterologous viruses and 10 negative donors were tested.

Our experiments and analyses demonstrate that the PBNA for Lassa Gpc is a sensitive, accurate, reproducible, and specific serological test for assessing neutralising activity against LASV. The results presented also highlight the importance of selecting a highly responsive control to establish all the acceptance criteria for assay validation, to determine relative accuracy and linearity. In fact, it was observed that while all 3 selected sera of the panel allowed to obtain an accurate and reproducible assay at all tested dilutions, the low serum 20/222 only met the acceptable criteria for accuracy and linearity from 1 to 8-fold dilutions.

It is important to emphasise that the PBNA-GPC Lassa assay presented can be considered a safe, reproducible and sensitive assay: safe because the pseudotypes are replication-deficient viruses containing LASSA-GP protein glycoproteins, devoid of virulent viral components and involved in a single round of replication; highly sensitive because we have used Nluc as a reporter gene to obtain a wide dynamic range of neutralisation titres; and reproducible because the reading was obtained by luminometer rather than a manual reading under the microscope dependent on operator interpretation. ^46^

In conclusion, the proposed validated PBNA-GP assay represents a powerful and safe platform for measuring the induction of the immune response in the detection of neutralising antibodies generated in response to the LASV vaccine antigen in vaccine evaluation. The data from this validation can be used to harmonise data between laboratories and the different vaccines in development thanks to the 1st International Reference Panel for Anti-Lassa Fever Virus Antibodies, providing a strong boost to progress in vaccine development against LASV.

## Ethical considerations

Not applicable.

## Patient consent

Not applicable.

## Permission to reproduce material from other sources

Not applicable.

## Clinical trial registration

Not applicable.

## Data Availability

The data that supports the findings of this study **“A validate and standardized pseudotyped microneutralization assay as a safe and powerful tool to measure LASSA neutralising antibodies for vaccine development and comparison.”** are available in biostudies portal AccessionS-BSST1413. DOI: 10.6019/S-BSST1413. ^47^ This project contains following dataset:
1. Validation table pseudotypes microneutralization assay. Data are available under the terms of the Creative Commons Zero “No rights reserved” data waiver (CC0 1.0 Public domain dedication).
