# Supplementary material for: A validated and standardized pseudotyped microneutralization assay as a safe and powerful tool to measure LASSA virus neutralising antibodies for vaccine development and comparison
Source: F1000Res. 2024 Oct 14;13:534. Originally published 2024 May 24. [Version 2] doi: 10.12688/f1000research.149578.2 (PMC11541077; doi:10.12688/f1000research.149578.2)
Supplement: Peer review history

# Reviewer report: Reviewer response for version 2

**DOI:** 10.5256/f1000research.172528.r331606

**Published:** 2024-11-06

**Authors:** Jinwoo Lee

**Affiliations:** 1 Department of Chemistry and Biochemistry, University of Maryland College Park, College Park, MD, USA

The authors addressed the concerns raised by the reviewer. However, some figures could be improved in resolution and it might be better to include a supplementary methods section with more details.

Is the work clearly and accurately presented and does it cite the current literature?

Yes

If applicable, is the statistical analysis and its interpretation appropriate?

Partly

Are all the source data underlying the results available to ensure full reproducibility?

Yes

Is the study design appropriate and is the work technically sound?

Yes

Are the conclusions drawn adequately supported by the results?

Yes

Are sufficient details of methods and analysis provided to allow replication by others?

Yes

Reviewer Expertise:

viral fusion

I confirm that I have read this submission and believe that I have an appropriate level of expertise to confirm that it is of an acceptable scientific standard.

# Reviewer report: Reviewer response for version 1

**DOI:** 10.5256/f1000research.164058.r299459

**Published:** 2024-09-13

**Authors:** Jinwoo Lee

**Affiliations:** 1 Department of Chemistry and Biochemistry, University of Maryland College Park, College Park, MD, USA

Overview:

 Antonelli et al. describe a method utilizing the pseudotyped microneutralization assay for testing antibodies in vaccine development. This study demonstrates that highly infectious viruses such as Lassa, Ebola, and Coronaviruses can be adapted for testing antibody activity using pseudotyped. The importance of applying pseudotyped viruses and validating the data is evident, as this method provides a safer and more accessible means to study these viruses, facilitating antibody testing in settings with limited biosafety facilities. Addressing the suggested improvements in figures, explanations, title, discussion, data representation, and abbreviations will enhance reader comprehension and the impact of the validation and standardization.

1. The figures in the article require improvement, and additional explanations should be added to enhance readers' understanding. Please revise the figures for better clarity and ensure they include comprehensive legends. The addition of detailed explanations in the results or discussion sections will help readers interpret the data more effectively since, for general readers, it might require a bit less explanation to digest the article thoroughly.
2. The title and discussion sections do not clearly explain how this method will be used for vaccine development. Please provide explicit connections in the discussion on how the pseudotyped microneutralization assay advances vaccine development. This can include examples of potential applications and anticipated outcomes.
3. The introduction contains conflicting information regarding death rates and case fatality rates (CFR). It's important to clarify the difference between the death rate of 5,000 out of 100,000-300,000 cases (<5%) and the CFR of around 20-30%. This will help readers understand the context and significance of each percentage, avoiding confusion and ensuring accurate interpretation of the data.
4.  Some data points in the figures are missing error bars or are too tight to be visible. For example, Figure 2B does not have any error bars, it might be helpful to add a standard error of the means.  Please review and adjust the figure settings as necessary to improve visibility.
5.  In Table 1, the observed GMT is higher than expected after dilution, with an observed GMT of always 10 after certain dilutions. It's important to provide an explanation for this observed trend in GMT after dilution. Discussing potential reasons for seeing a GMT of 10 only at high dilutions will help readers understand the data and its implications. Furthermore, relative accuracy is failed and passed again when it is diluted.  The authors explanation will help readers.
6. Some abbreviations are not spelled out the first time they are used. Please ensure all abbreviations are spelled out when first used in the text. This will help readers who may not be familiar with the terminology.
7. When pseudoparticles are produced, the shape of the particle might be more influenced by the matrix protein. To expand this method, it might be useful to suggest that glycoprotein is more important than the shape and size of the actual virus for the binding of antibodies, considering that Ebola is a filovirus.

Is the work clearly and accurately presented and does it cite the current literature?

Yes

If applicable, is the statistical analysis and its interpretation appropriate?

Partly

Are all the source data underlying the results available to ensure full reproducibility?

Yes

Is the study design appropriate and is the work technically sound?

Yes

Are the conclusions drawn adequately supported by the results?

Yes

Are sufficient details of methods and analysis provided to allow replication by others?

Yes

Reviewer Expertise:

viral fusion

I confirm that I have read this submission and believe that I have an appropriate level of expertise to confirm that it is of an acceptable scientific standard, however I have significant reservations, as outlined above.

# Response

**Published:** 2024-09-27

**Authors:** roberta antonelli

**Affiliations:** vismederi, Italy

**Jinwoo Lee**

 1_ ***The figures in the article require improvement, and additional explanations should be added to enhance readers' understanding. Please revise the figures for better clarity and ensure they include comprehensive legends.***

 The addition of detailed explanations in the results or discussion sections will help readers interpret the data more effectively since, for general readers, it might require a bit less explanation to digest the article thoroughly.

 To improve the quality of the article figure legends have been introduced in all figures, some details or paragraph have been introduced in the Introduction and Discussion, regarding the results some details are yet elucidated in the Materials and Methods section because we would like to maintain the compact structure of the paper.

 2_ ***The title and discussion sections do not clearly explain how this method will be used for vaccine development. Please provide explicit connections in the discussion on how the pseudotyped microneutralization assay advances vaccine development. This can include examples of potential applications and anticipated outcomes.***

 To emphasise that pseudotyped neutralisation assays can be extremely useful in assessing not only how well circulating virus isolates match vaccine formulations, but also how well these vaccines can cross-protect against emerging drift variants, a paragraph has been added to the Discussion section. In this section, we have cited several examples where the applicability of pseudotyped LV-based neutralisation assays has been demonstrated as a useful tool for investigating NAbs generated by different vaccine candidates.

 3_ ***The introduction contains conflicting information regarding death rates and case fatality rates (CFR). It's important to clarify the difference between the death rate of 5,000 out of 100,000-300,000 cases (<5%) and the CFR of around 20-30%. This will help readers understand the context and significance of each percentage, avoiding confusion and ensuring accurate interpretation of the data.*** We have rewritten this point more clearly adding some sentences in the introduction section

 3_ ***Some data points in the figures are missing error bars or are too tight to be visible.*** **For example, Figure 2B does not have any error bars, it might be helpful to add a standard error of the means.  Please review and adjust the figure settings as necessary to improve visibility.**

 We have introduced the error bars and the figures 2 , 5 and 6 are reuploaded

   4_ ***In Table 1, the observed GMT is higher than expected after dilution, with an observed GMT of always 10 after certain dilutions. It's important to provide an explanation for this observed trend in GMT after dilution. Discussing potential reasons for seeing a GMT of 10 only at high dilutions will help readers understand the data and its implications. Furthermore, relative accuracy is failed and passed again when it is diluted.  The authors explanation will help readers.***

 As explain in the paper The accuracy of the test was evaluated by using the reportable (RP) values obtained during the evaluation of Linearity. Following the ICH guideline the accuracy can be tested by using either a conventional true value or an accepted reference value. The GMT of the expected values was calculated from the GMT of the results obtained from the neat sample, and by dividing this value by the corresponding factor of the twofold serial dilution. Relative accuracy was evaluated by calculating the percentage of recovery on the GMT of the RP values and the expected (true) titer and applying the formula: 100 × (GMT observed/GMT expected). Is it true that in one point of the 20/ 228 the observed GMT is higher than expected but follow the *criteria*

**The assay is considered to have acceptable relative accuracy if the percentage of recovery on the GM of the reportable values and the expected is within 50% - 200%.**

***5_Some abbreviations are not spelled out the first time they are used. Please ensure all abbreviations are spelled out when first used in the text. This will help readers who may not be familiar with the terminology***.

 We have introduced these changes.

**6_When pseudoparticles are produced, the shape of the particle might be more influenced by the matrix protein.**

 We have produced the Lassa pseudotype virus using GAG from HIV and this pseudotype is predominantly spherical in shape when in-vitro.

***To expand this method, it might be useful to suggest that glycoprotein is more important than the shape and size of the actual virus for the binding of antibodies, considering that Ebola is a filovirus.***

 We agree with the reviewer that the GP is more important than the shape and size of the actual virus for the binding of antibodies. We are aware of the fact that for some viruses, like Ebola, that it may be preferable to pseudotype the GP onto a virus core with a more comparable morphology such as a rhabdoviral VSV core. However in this manuscript we are working with Lassa virus which has a spherical structure making it highly compatible with the HIV core used.

# Reviewer report: Reviewer response for version 1

**DOI:** 10.5256/f1000research.164058.r299463

**Published:** 2024-07-16

**Authors:** Jesse Bloom, Caleb Carr

**Affiliations:** 1 University of Washington, Seattle, WA, USA

**SUMMARY:**

 This paper describes a pseudotyped lentivirus neutralization assay for antibodies targeting Lassa virus GPC. Such an assay is useful for characterization of immunity, vaccines, and antibodies to this virus. This paper is a useful addition to the literature and has the scientific content to merit publications.

 However, there are a number of small inconsistencies in phrasing or other small oversights/errors that should be addressed through minor modifications in the final version. These minor critiques are listed below:

**SPECIFIC COMMENTS:**

 - Title should say "validated" not "validate"

 - Title should say "Lassa virus" not "LASSA"

 - Abstract: there is a typo where it says "LSV" rather than "LASV"

 - Introduction: in the paragraph beginning "LASV case fatality rates (CFRs) differ between countries..." there is some sort of typo where it says the CFR is "69%65". More generally, case fatality rates can depend heavily on the efficiency of how many non-severe infections are detected as cases, so this comparison may reflect more about the case ascertainment in different countries than the actual severity of disease following infection.

 - In the paragraph beginning "Improvement in the development of Lassa vaccine and treatments has been hampered..." it might be better to say "complicated" rather than "hampered". The word "hampered" implies it is a bad thing that Lassa is a BSL-4 pathogen, but there are good reasons for this classification given the severity of the virus.

 - Citations should be made to prior work that has pseudotyped LASV GPC using methods similar to those in this paper. For instance, see the following PMIDs: **(**Carr et al.,2024), (Larson et al.,2004), (Reignier et al.,2006), (Kunz et al.,2005)

 - Figure 1: I assume "CC CTR" refers to some type of negative control, but the figure legend does not provide an explanation of what that means. This should be explained.

 - The paper should provide more details on the five anti-LASV reference sera including what they represent and how they were obtained.

 - The images in Figure 2 are very low resolution and clipped on the right side.

 - First paragraph of the discussion: this claim seems dubious; is there actually evidence that COVID-19 increased occurrences of Lassa fever? Lassa was causing infections long before COVID-19 and the two seem largely unrelated.

 - Second sentence of Discussion: should be "increased" not "improved"

 - Second-to-last paragraph of Discussion: should be "LASV GPC" not "LASSA-GP"

 - Methods: Incomplete sentence in section 2.3: “For LASV pseudotype production, approximately 4.5x106 HEK293T/17 cells.”

 - Discussion: five instances of typo where it says “LSV” instead of “LASV”

 - Figures 3, 5, and 6 do not have legends. Adding legends will help with interpreting the plots

 - Figure 6 has the following typos "Standars agaist other infection" and "Negative serum F pannel"

Is the work clearly and accurately presented and does it cite the current literature?

Partly

If applicable, is the statistical analysis and its interpretation appropriate?

Yes

Are all the source data underlying the results available to ensure full reproducibility?

Yes

Is the study design appropriate and is the work technically sound?

Yes

Are the conclusions drawn adequately supported by the results?

Yes

Are sufficient details of methods and analysis provided to allow replication by others?

Partly

Reviewer Expertise:

Virus evolution

We confirm that we have read this submission and believe that we have an appropriate level of expertise to confirm that it is of an acceptable scientific standard.

## References

[1] : Deep mutational scanning reveals functional constraints and antigenic variability of Lassa virus glycoprotein complex. *bioRxiv* .2024; 10.1101/2024.02.05.579020 10.1101/2024.02.05.579020 39013466 PMC11390330

[2] : Identification of a broad-spectrum arenavirus entry inhibitor. *J Virol* .2008;82(21) : 10.1128/JVI.00941-08 10768-75 10.1128/JVI.00941-08 18715909 PMC2573164

[3] : Receptor use by pathogenic arenaviruses. *Virology* .2006;353(1) : 10.1016/j.virol.2006.05.018 111-20 10.1016/j.virol.2006.05.018 16797051

[4] : Characterization of the interaction of lassa fever virus with its cellular receptor alpha-dystroglycan. *J Virol* .2005;79(10) : 10.1128/JVI.79.10.5979-5987.2005 5979-87 10.1128/JVI.79.10.5979-5987.2005 15857984 PMC1091707

# Response

**Published:** 2024-09-27

**Authors:** roberta antonelli

**Affiliations:** vismederi, Italy

**Jesse D Bloom**

 SPECIFIC COMMENTS:

 - ***Title should say "validated" not "validate"***  

  the correction has been incorporated

 - ***Title should say "Lassa virus" not "LASSA" ***.

 the correction has been incorporated

 - ***Abstract: there is a typo where it says "LSV" rather than "LASV" *** 

 the correction has been incorporated

 - I ***ntroduction: in the paragraph beginning "LASV case fatality rates (CFRs) differ between countries..." there is some sort of typo where it says the CFR is "69%65". More generally, case fatality rates can depend heavily on the efficiency of how many non-severe infections are detected as cases, so this comparison may reflect more about the case ascertainment in different countries than the actual severity of disease following infection.***

 The paragraph was rewritten

 - ***In the paragraph beginning "Improvement in the development of Lassa vaccine and treatments has been hampered..." it might be better to say "complicated" rather than "hampered". The word "hampered" implies it is a bad thing that Lassa is a BSL-4 pathogen, but there are good reasons for this classification given the severity of the virus.***

 the correction has been incorporated

 - ***Citations should be made to prior work that has pseudotyped LASV GPC using methods similar to those in this paper. For instance, see the following PMIDs: (Carr et al.,2024), (Larson et al.,2004), (Reignier et al.,2006), (Kunz et al.,2005)***

 the references has been incorporated

 - ***Figure 1: I assume "CC CTR" refers to some type of negative control, but the figure legend does not provide an explanation of what that means.***

 Explanation in the figure legend has been incorporated

 - ***The paper should provide more details on the five anti-LASV reference sera including what they represent and how they were obtained***.

 In the paragraph 3.2 we have explained that is a panel from MHRA Lassa panel 21/332 established by an expert committed . In the material and method and in the main , we introduced the detail of this WHO International Reference Panel for anti-Lassa fever virus antibodies  NIBSC code: 21/332. This panel is commercially available from NIBCShttps://nibsc.org/products/brm_product_catalogue/detail_page.aspx?catid=21/332

 - ***The images in Figure 2 are very low resolution and clipped on the right side.***

 We have improved the image quality

 - ***First paragraph of the discussion: this claim seems dubious; is there actually evidence that COVID-19 increased occurrences of Lassa fever? Lassa was causing infections long before COVID-19 and the two seem largely unrelated.***

 The sentence was rewritten

 - ***Second sentence of Discussion: should be "increased" not "improved"***

 The change has been made the correction has been incorporated

 - S ***econd-to-last paragraph of Discussion: should be "LASV GPC" not "LASSA-GP*** "

 the correction has been incorporated

 - ***Methods: Incomplete sentence in section 2.3: “For LASV pseudotype production, approximately 4.5x106 HEK293T/17 cells.”***

 the correction has been incorporated

 - ***Discussion: five instances of typo where it says “LSV” instead of “LASV”***

 he corrections have been incorporated

 - ***Figures 3, 5, and 6 do not have legends. Adding legends will help with interpreting the plots***

 the legends have been incorporated

 - ***Figure 6 has the following typos "Standars agaist other infection" and "Negative serum F pannel"***

 the correction has been incorporated

# Response

**Published:** 2024-09-27

**Authors:** roberta antonelli

**Affiliations:** vismederi, Italy

SPECIFIC COMMENTS:

 - ***Title should say "validated" not "validate"***  

  the correction has been incorporated

 - T ***itle should say "Lassa virus" not "LASSA" .***

 the correction has been incorporated

 - A ***bstract: there is a typo where it says "LSV" rather than "LASV"***

******the correction has been incorporated

 - ***Introduction: in the paragraph beginning "LASV case fatality rates (CFRs) differ between countries..." there is some sort of typo where it says the CFR is "69%65". More generally, case fatality rates can depend heavily on the efficiency of how many non-severe infections are detected as cases, so this comparison may reflect more about the case ascertainment in different countries than the actual severity of disease following infection.***

 The paragraph was rewritten

 - I ***n the paragraph beginning "Improvement in the development of Lassa vaccine and treatments has been hampered..." it might be better to say "complicated" rather than "hampered". The word "hampered" implies it is a bad thing that Lassa is a BSL-4 pathogen, but there are good reasons for this classification given the severity of the virus.***

 the correction has been incorporated

 - ***Citations should be made to prior work that has pseudotyped LASV GPC using methods similar to those in this paper. For instance, see the following PMIDs: (Carr et al.,2024), (Larson et al.,2004), (Reignier et al.,2006), (Kunz et al.,2005)***

 the references has been incorporated

 - ***Figure 1: I assume "CC CTR" refers to some type of negative control, but the figure legend does not provide an explanation of what that means***.

 Explanation in the figure legend has been incorporated

 - ***The paper should provide more details on the five anti-LASV reference sera including what they represent and how they were obtained.***

 In the paragraph 3.2 we have explained that is a panel from MHRA Lassa panel 21/332 established by an expert committed . In the material and method and in the main , we introduced the detail of this WHO International Reference Panel for anti-Lassa fever virus antibodies  NIBSC code: 21/332. This panel is commercially available from NIBCShttps://nibsc.org/products/brm_product_catalogue/detail_page.aspx?catid=21/332

 - ***The images in Figure 2 are very low resolution and clipped on the right side.***

 We have improved the image quality

 - ***First paragraph of the discussion: this claim seems dubious; is there actually evidence that COVID-19 increased occurrences of Lassa fever? Lassa was causing infections long before COVID-19 and the two seem largely unrelated.***

 The sentence was rewritten

 - ***Second sentence of Discussion: should be "increased" not "improved"***

 The change has been made the correction has been incorporated

 - ***Second-to-last paragraph of Discussion: should be "LASV GPC" not "LASSA-GP"***

 the correction has been incorporated

 - ***Methods: Incomplete sentence in section 2.3: “For LASV pseudotype production, approximately 4.5x106 HEK293T/17 cells***.

 ” the correction has been incorporated

 - ***Discussion: five instances of typo where it says “LSV” instead of “LASV”***

 the corrections have been incorporated

 - ***Figures 3, 5, and 6 do not have legends. Adding legends will help with interpreting the plots the*** legends have been incorporated

 - **Figure 6 has the following typos "Standars agaist other infection" and "Negative serum F pannel"**

 the correction has been incorporated
